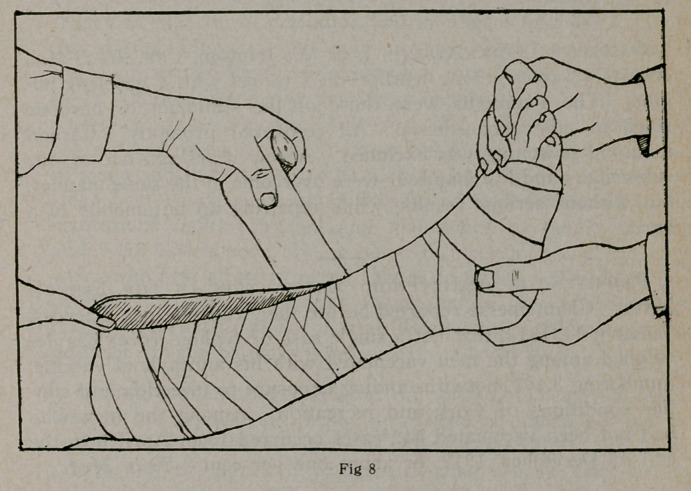# Plastic Splints for General Use, with a Special Description of the Application of Leg Splints

**Published:** 1913-09

**Authors:** J. J. Buchanan

**Affiliations:** Surgeon to Mercy Hospital, Pittsburgh, Pa.


### BUFFALO MEDICAL JOURNAL

VOLUME 69 SEPTEMBER, 1913 No. 2

### ORIGINAL ARTICLES

*No responsibility is assumed by the Journal for the opinions of contributors of original articles; nor for methods of expression nor errors in proof reading, excepting for articles in a foreign language contributed for translation. The right is reserved to decline contributions on account of bona fide lack of space; or because they do not bear on practical medicine and surgery; or because they would fail to interest or would give offense to the majority of subscribers. The expression of minority views or reasonable and courteous criticism of the views of others is not considered offensive.*

### Plastic Splints For General Use, With a Special Description of the Application of Leg Splints

BY J. J. BUCHANAN, M. D.
Surgeon to Mercy Hospital, Pittsburgh, Pa.

EIGHTEEN years ago the writer devised a plastic splint of Crinoline, plaster of Paris and Lintine, which has been used continuously since that time by his surgical colleagues and himself, in the Mercy Hospital, to the almost entire exclusion of all other splint materials.

Fig. 1

In the year 1896, he described the splint at the Harrisburg meeting of the Medical Society of the State of Pennsylvania, and this description will be found in the transactions of the Society for that year (pages 59 and 60.)

A description of the splint is also to be found in Dr. Nicholas Senn's work on "Practical Surgery" (pages 547 et seq.) published in 1901.

Fig. 2

Notwithstanding the publicity given the method by the work of Professor Senn, the writer considers that the present additional

Fig. 3

publication is justified as an effort to bring the advantages of this method of making splints to the notice of the profession.

The illustrations herewith given are of leg splints only, and they are the only ones kept constantly in reserve; but in fractures of the arm, forearm and femur (in the last, only where consolidation is advanced) ; in immobilization of joints for inflammatory and tubercular conditions and after excision of joints, reduction of dislocations and in other joint injuries, the employment of splints of the same kind in appropriate shapes has been practically invariable.

The splint proper consists of a body of six to eight layers of crinoline, into the meshes of which plaster of Paris has been rubbed, with a facing and back of lintine, which is a thin, smooth-

Fig. 4

surfaced sheet of compressed cotton. Leg splints are in such daily requirement and are of such uniform size that it has been found desirable to have considerable numbers of them in readiness at all times; but splints for other parts are made to order in exactly the same way, of such size and shape as the case may demand.

*Preparation of the Splint.*—Cut the required number (6 to 8) of pieces of crinoline of the size and shape of the splint desired. Cut two pieces of lintine of the same size and shape. Lay one of the crinoline shapes on a table and rub plaster of Paris well into its meshes. Lay on it exactly another crinoline shape and rub its meshes full. Repeat this till the requisite number of layers of crinoline are together.

Figure 1 shows two sets of the manifold crinoline shapes, four lintine shapes and three gauze bandages with a tin container. These are all the materials necessary for the application of

Fig. 5

two lateral leg splints and, kept in the box to exclude moisture, they may be kept indefinitely, ready for instant use. This outfit is advised for private practice on account of its portability.

*Application of the Splint.*—Remove the manifold crinoline shape from its box and hold it in its folded condition in a basin

Fig. 6

of water, taking care to shake out as little as possible of the plaster either in handling or in soaking. When the bubbles have ceased to rise, lift the crinoline gently from the water and

Fig. 7

squeeze out the excess of water, as shown in Figure 2. Spread the shape on a table with the hands, as shown in Figure 3, removing all wrinkles and causing it to resume its original form.

Fig 8

Lift the plastic shape from the table and lay it on one of the lintine shapes, as shown in Figure 4, pressing it down at every part with the hands. Lay another lintine shape on the plastic shape and press all well together with the hands. Trim uneven edges, as shown in Figure 5.

The splint is then ready for application to the limb and, as shown in Figure 6, is as pliable as a wet wash-rag, and back, front and body are as one piece. The limb is held in the desired position, the splint applied accurately to all its inequalities, without any padding whatever, and bound to the limb with a smoothly applied roller bandage, as shown in Figure 7.

The second splint is made and applied to the other aspect of the limb with a second roller, as appears in Figure 8.

The splints, of course, soon harden, and when removed they are light, firm, clean, accurately fitting and easily reapplied. With their use, pressure sores are unknown and pads both unnecessary and inadvisable.

When the splints have been removed for any purpose, they should be reapplied in the same manner and order. Redness over a salient point indicating pressure should never be met by padding, but by making a fenestra in the splint opposite the reddened area. This is very rarely necessary. When the part shrinks, so that the splints fit imperfectly, new ones should always be applied. This is often necessary in the later course of leg fractures.

---

Gasolene Intoxication. J. G. W. Johnson, *Can. Med. Mo.*, reports 42 cases among workers in a tunnel with a gasolene engine. The symptoms were those of the stimulant or narcotic stage of ether anaesthesia. All recovered promptly. Carbon monoxid poisoning was excluded. A few years ago one of our subscribers and his chauffeur were overcome in the same manner, but without serious results, while repairing an automobile in a barn.

---

Prophylactic Antityphoid Vaccination in the French Navy. Chantemesse reported before the Academie des Sciences, January 20, 1913, that not a single case of typhoid fever has developed among the men vaccinated with his antitpyhoid vaccine, numbering 3,107, notwithstanding exposure to infection and similar conditions of work and recreation. Among the men who had not been vaccinated 542 cases occurred from April 5 to the end of December, 1912, or about one per cent.—*Trib. Med.*

## Figures and Tables

**Fig. 1 f1:**
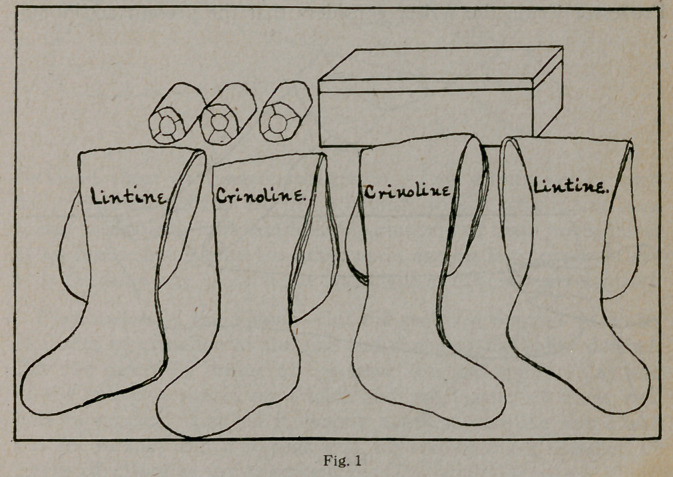


**Fig. 2 f2:**
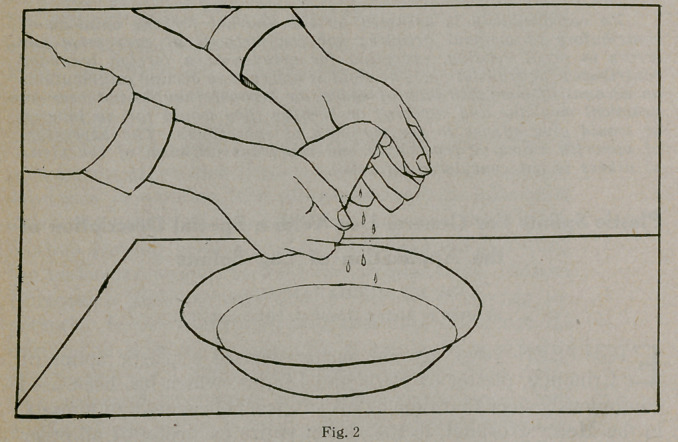


**Fig. 3 f3:**
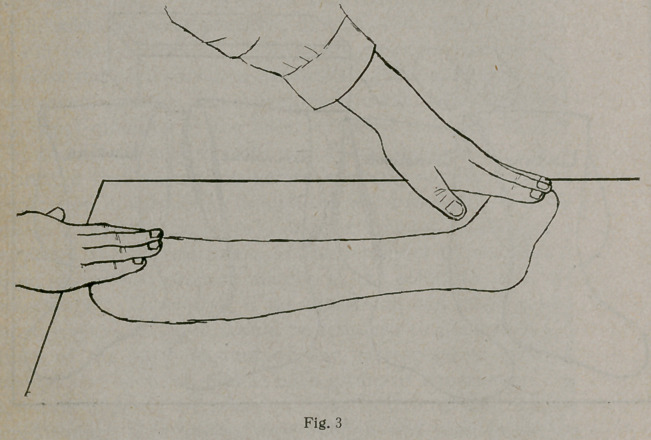


**Fig. 4 f4:**
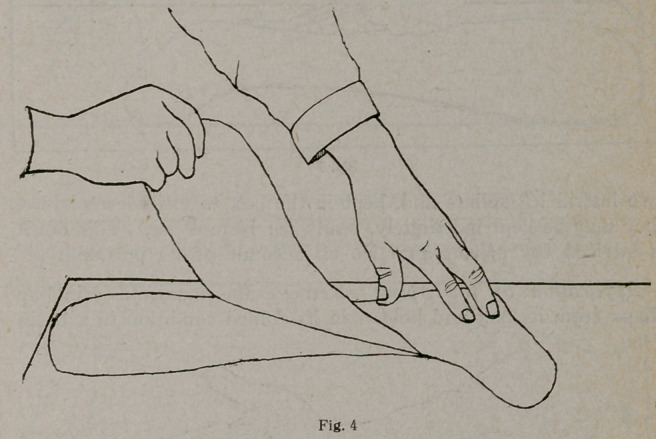


**Fig. 5 f5:**
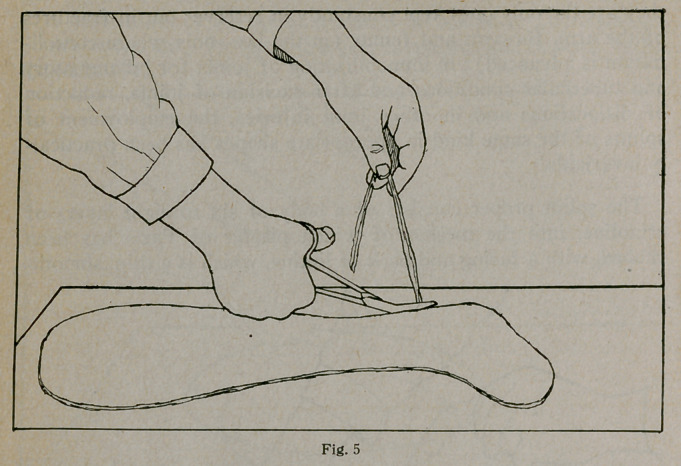


**Fig. 6 f6:**
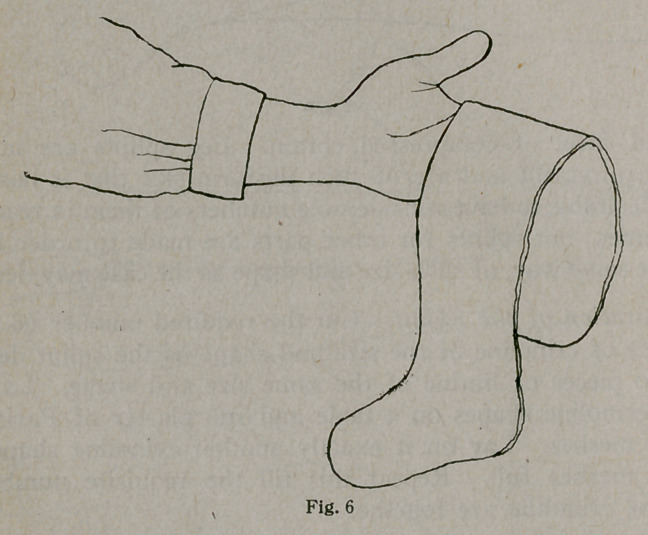


**Fig. 7 f7:**
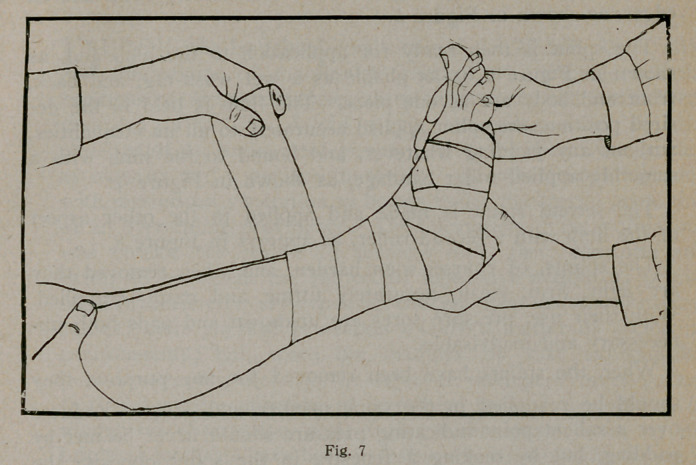


**Fig. 8 f8:**